# Epidermal stem cells maintain stemness via a biomimetic micro/nanofiber scaffold that promotes wound healing by activating the Notch signaling pathway

**DOI:** 10.1186/s13287-021-02418-2

**Published:** 2021-06-10

**Authors:** Zhixiao Lin, Congying Zhao, Zhanjun Lei, Yuheng Zhang, Rong Huang, Bin Lin, Yuchen Dong, Hao Zhang, Jinqing Li, Xueyong Li

**Affiliations:** Department of Plastic Surgery, Tangdu Hospital, Airforce Military Medical University, Xi’an, 710038 China

**Keywords:** Epidermal stem cell, Micro/nanofiber scaffold, Cell differentiation, Wound healing, Notch pathway

## Abstract

**Background:**

Epidermal stem cells (EpSCs) play a vital role in wound healing and skin renewal. Although biomaterial scaffolds have been used for transplantation of EpSCs in wound healing, the ex vivo differentiation of EpSCs limits their application.

**Methods:**

To inhibit the differentiation of EpSCs and maintain their stemness, we developed an electrospun polycaprolactone (PCL)+cellulose acetate (CA) micro/nanofiber for the culture and transplantation of EpSCs. The modulation effect on EpSCs of the scaffold and the underlying mechanism were explored. Liquid chromatography-tandem mass spectrometry for label-free quantitative proteomics was used to analyze proteomic changes in EpSCs cultured on scaffolds. In addition, the role of transplanted undifferentiated EpSCs in wound healing was also studied.

**Results:**

In this study, we found that the PCL+CA micro/nanofiber scaffold can inhibit the differentiation of EpSCs through YAP activation-mediated inhibition of the Notch signaling pathway. Significantly differentially expressed proteomics was observed in EpSCs cultured on scaffolds and IV collagen-coated culture dishes. Importantly, differential expression levels of ribosome-related proteins and metabolic pathway-related proteins were detected. Moreover, undifferentiated EpSCs transplanted with the PCL+CA scaffold can promote wound healing through the activation of the Notch signaling pathway in rat full-thickness skin defect models.

**Conclusions:**

Overall, our study demonstrated the role of the PCL+CA micro-nanofiber scaffold in maintaining the stemness of EpSCs for wound healing, which can be helpful for the development of EpSCs maintaining scaffolds and exploration of interactions between biomaterials and EpSCs.

## Background

Epidermal stem cells (EpSCs) are an important component of the epidermis and play a vital role in wound healing and skin renewal through proliferation and terminal differentiation [1]. The attachment of EpSCs to the basement membrane is associated with changes in cytoskeletal remodeling and cell morphology that preserve the stemness and proliferation ability of EpSCs and inhibit differentiation [2]. As EpSCs lose adhesion with the basement membrane and enter the upper layers of stratified epithelium, they initiate the terminal differentiation program until they are finally shed from skin surfaces [3, 4]. Maintaining the stemness of EpSCs remains a difficulty that limits the use of EpSCs in wound healing and other EpSC-related diseases [5, 6]. Therefore, finding ways to maintain the stemness of EpSCs and further elucidating the underlying mechanism are urgent and necessary.

Electrospinning is recognized as a versatile and multifunctional technique to establish fibrillar structures with nano/microscale fineness [7, 8]. The electrospun nanofiber scaffold can closely resemble the interwoven fiber meshwork of collagen and elastic fibers. For the use of nanofiber scaffolds in modulating cell differentiation, researchers used a sericin-loaded cationic gelatin-hyaluronan-chondroitin sulfate nanofiber scaffold to induce the directional differentiation of human mesenchymal stem cells into epithelial cells [9]. In our previous study, we used a PCL+CA+CS trilayer micro/nanofiber scaffold to reconstruct the basement membrane in vitro, which shows the potential of micro/nanofiber structure scaffolds in mimicking the in vivo biological environment [10]. The differentiation of EpSCs is modulated by the extracellular microenvironment. Thus, we speculated that micro/nanofiber scaffolds fabricated by electrospinning can maintain the undifferentiated status of EpSCs, which is also recognized as the stemness of EpSCs.

YAP and TAZ are important for the transduction of cell-sensed mechanical signals into cellular contexts [11, 12]. Of note, mechanically dependent regulation of YAP/TAZ can regulate the level of the Notch pathway [13]. In the epidermis, activation of the Notch pathway is vital in the differentiation of EpSCs and in turn promotes wound healing in vivo [14]. Thus, inhibition of the Notch pathway is needed to inhibit the differentiation of EpSCs to maintain stemness in vitro.

Elucidating interactions between EpSCs and the extracellular microenvironment is important. However, exploring individual proteins within the massive proteins in EpSCs is difficult. The application of proteomic techniques provides tools for exploration. Liquid chromatography-tandem mass spectrometry (LC-MS/MS) for label-free quantitative proteomics (LFQP) is an important MS tool to detect and quantify large amounts of proteins [15]. Herein, we used LFQP to determine potentially different proteins in EpSCs cultured in scaffold environments and normal culture substrate environments and reveal the possible mechanisms underlying the modulation effect of micro/nanofiber scaffolds on EpSCs.

In our study, we explored the role of the PCL+CA micro/nanofiber scaffold in maintaining the stemness of EpSCs for wound healing, which can be helpful for the development of EpSCs maintaining scaffolds in the clinic and exploration of interactions between biomaterials and EpSCs.

## Materials and methods

### Materials

Poly(ε-caprolactone) (PCL, *M*_*W*_ = 70-90 KDa) and cellulose acetate (CA, *M*_*n*_ = 30 KDa) were purchased from Sigma-Aldrich Chemistry (USA). All other chemicals were of analytical grade and used as received. Sprague–Dawley (SD) were obtained from the Experimental Animal Center of Airforce Military Medical University and kept under standard conditions according to the regulation of ethics committee of the Medical Sciences Department.

### Isolation and culture of EpSCs

EpSCs were isolated from the backs of newborn (0–3 days) SD rat skin. Briefly, skin samples were taken from the backs of SD rats and cut into pieces (0.2×0.3 cm). After incubation in 0.1% Dispase II in PBS at 4°C overnight, the epidermal sheet was carefully separated from the dermis, minced, and then digested in 0.25% trypsin in PBS for 10 min at 37°C. Trypsin was inactivated in calcium-free RPMI 1640 medium containing 10% FBS. Following filtering and centrifugation, the cells were resuspended in keratinocyte serum-free medium (KM, #2101; ScienCell) and seeded at a density of 10^5^ cells/cm^2^ in flasks coated with 100 μg/ml collagen IV (C5533; Sigma) to adhere for 20 min at 37°C. The nonadherent cells were subsequently discarded, and rapidly adhering cells were cultured in KM at 37°C in 5% CO_2_. Meanwhile, the cells were identified as EpSCs with CK19^bri^ (10712-1-AP; Proteintech), CD34^bri^ (ab119695; Abcam), and CD71^dim^ by flow cytometry (data not shown).

### Preparation of electrospun fibers

The 8 wt% PCL+14 wt% CA blend solution was prepared as reported [13]. For the preparation of the PCL+CA micro/nano scaffold, a collector distance of 15 cm at a feed rate of 1 mL/h through a flat-tip stainless steel spinneret (Ø = 0.4 × 25 mm) for the microfiber and a collector distance of 20 cm at a feed rate of 0.5 mL/h through a spinneret (Ø = 0.2 × 25 mm) for the nanofiber assisted by a programmable syringe pump (LSP02-1B, Baoding Longer Precision PumpCo., Ltd, China) were used. The DC voltage was 15 kV for the micro fiber and 21kV for the nanofiber. To avoid contamination, membranes were prepared on a clean bench and air-dried under laminar air flow after being incubated in ethanol (70% v/v) for 10 min. Then, they were punched into round sections and sterilized by ethylene oxide gas supplied by the disinfection and supply the Department of Tangdu Hospital prior to further characterization and cell culture experiments.

### Characterization of micro/nanofiber scaffold

Briefly, the structural morphology of electrospun fibers was analyzed by cold-type FE-SEM (FE-SEM, JSM-6700F, JEOL, Japan). Samples were cut into small pieces (1 cm × 1 cm), adhered to a stainless steel sample holder and sputter coated with gold. The specimens were then examined using FE-SEM at 5 kV. The average fiber size was determined by measuring the diameters of 100 nanofibers using Photoshop 7.0 edition. Stress-strain curve of the scaffold was tested by tensile strength tester (Instron 5566, Illinois Tool Works, USA). Other tests of the PCL+CA scaffold, such as determination of composition, were documented in our previously published articles [16].

### Western blot analysis

Cells and skin tissues were lysed with Western lysis buffer [150 mM NaCl, 0.1% NP-40, 0.5% sodium deoxycholate, 50 mM Tris (pH 8.0)] supplemented with complete protease inhibitor cocktail (Roche), and the protein content was determined by a Bradford assay. Then, cell proteins were separated with 10% SDS-PAGE and transferred onto PVDF membranes for hybridization. After blocking, the PVDF membranes were incubated with the primary antibodies overnight at 4°C. After washing in TBST [50 mM Tris (pH 8), 150 mM NaCl, and 0.05% Tween 20] 3 times, the membrane was incubated with secondary antibody conjugated to horseradish peroxidase (HRP) for 1 h at room temperature (detailed information on the antibodies is listed in Table 1). Bands were visualized with electrochemical luminescence (ECL) solution and the Bio-Rad Image Lab system v3.0. The densities of bands were measured with ImageJ software v1.53a (NIH, Bethesda, MD, USA).

**Table 1:** Detailed information on the antibodies for western blot analysis

Antibody	Dilution ratio	Source
Polyclonal anti-cyclin D1	1:1000	26939-1-AP; Proteintech
Polyclonal anti-cyclin E1	1:1000	11554-1-AP; Proteintech
Monoclonal[EP1607IHCY] Anti-cytokeratin 10	1:5000	ab76318; Abcam
Polyclonal anti-cytokeratin 14	1:2000	10143-1-AP; Proteintech
Polyclonal anti-cytokeratin 19	1:2000	10712-1-AP; Proteintech
Polyclonal anti-Notch1	1:1000	20687-1-AP; Proteintech
Monoclonal[D4Y1R] Anti-Jagged1	1:1000	#70109; CST
Monoclonal[D6P2U] Anti-Hes1	1:1000	#11988; CST
Polyclonal anti-GAPDH	1:5000	10494-1-AP; Proteintech
Polyclonal anti-rabbit IgG(H&L)	1:10000	A0208, Beyotime, China
Polyclonal anti-mouse IgG(H&L)	1:10000	A0192, Beyotime, China

### Histological analysis

For the histological study, tissues were fixed in formaldehyde, dehydrated, embedded in paraffin, and sectioned at 4 μm. The sectioned tissues were deparaffinized and stained with Masson and PAS. The skin tissue was examined and imaged in random order with standard light microscopy.

### Immunostaining for fluorescence microscopy

Cells cultured on confocal dishes or nanofiber scaffolds were rinsed with phosphate-buffered saline (PBS), fixed with 4% paraformaldehyde in PBS, washed with PBS 3 times, and permeated with Triton X-100 (0.25%) in PBS for 15 min. Cells or tissue sections were blocked in 2% BSA in PBS and then incubated with primary antibodies overnight at 4°C (Table 2). After washing with PBS three times, cells or tissue sections were incubated with conjugated secondary antibody at room temperature for 2 h. DAPI (Sigma Aldrich) was used at a 1:1000 dilution for nuclear staining. Cells or sections were viewed and imaged by using a fluorescence microscope (Leica Microsystems, DMIRB, Wetzlar, Germany).

**Table 2:** Detailed information on the antibodies for fluorescence microscopy

Antibody	Dilution ratio	Source
Polyclonal Anti-cytokeratin 19	1:200	10712-1-AP; Proteintech
Monoclonal[PC10] Anti-PCNA	1:200	Ab29; Abcam
Monoclonal[F-6] Anti-integrin ɑ6	1:150	sc-374057; Santa Cruz
Monoclonal[EPR16778] Anti-β-tublin	1:300	Ab179511; Abcam

### Liquid chromatography-tandem mass spectrometry for label-free quantitative proteomics

Protein samples were prepared and sent to the Shanghai Genechem Co., Ltd. for further liquid chromatography-tandem mass spectrometry for LFQP. First, the resulting peptide of each sample was prepared by filter-aided sample preparation digestion. Then, the peptide of each sample was desalted on C18 cartridges, concentrated by vacuum centrifugation, and reconstituted in 40 μl of 0.1% (v/v) formic acid. The peptide content was estimated by UV light spectral density at 280 nm using an extinction coefficient of 1.1 for a 0.1% (g/l) solution that was calculated on the basis of the frequency of tryptophan and tyrosine in vertebrate proteins. LC-MS/MS analysis was performed on a Q Exactive Plus mass spectrometer (Thermo Fisher Scientific) that was coupled to Easy nLC (Thermo Fisher Scientific). Two micrograms of peptide was loaded onto a C18 reversed-phase analytical column (Thermo Fisher Scientific, Acclaim PepMap RSLC 50 μm x 15 cm, nano viper, P/N164943) in buffer A (0.1% formic acid) and separated with a linear gradient of buffer B (80% acetonitrile and 0.1% formic acid) at a flow rate of 300 nl/min. The linear gradient was as follows: 5% buffer B for 5 min, 5–28% buffer B for 90 min, 28–38% buffer B for 15 min, 38–100% buffer B for 5 min, and holding in 100% buffer B for 5 min. MS data were acquired using a data-dependent top 10 method that dynamically chooses the most abundant precursor ions from the survey scan (350–1800 m/z) for HCD fragmentation. MS1 scans were acquired at a resolution of 70,000 at m/z 200 with an AGC target of 3e6 and a maxIT of 50 ms. MS2 scans were acquired at a resolution of 17,500 at m/z 200 with an AGC target of 2e5 and a maxIT of 45 ms, and the isolation width was 2 m/z. Only ions with a charge state between 2 and 6 and a minimum intensity of 2e3 were selected for fragmentation. Dynamic exclusion for selected ions was 30 s, and the normalized collision energy was 27 eV.

The MS data were analyzed using MaxQuant software version 1.6.14.0 and searched against the UniProt database. An initial search was set at a precursor mass window of 6 ppm. The search followed an enzymatic cleavage rule of trypsin/P and allowed two maximal missed cleavage sites and a mass tolerance of 20 ppm for fragment ions. Carbamidomethylation of cysteines was defined as a fixed modification, while protein N-terminal acetylation and methionine oxidation were defined as variable modifications for database searching. The cutoff of the global false discovery rate (FDR) for peptide and protein identification was set to 0.01. Protein abundance was calculated on the basis of the normalized spectral protein intensity (LFQ intensity). Proteins with a fold change >2 or <0.5 and a p value (Student’s t test) <0.05 were considered differentially expressed proteins.

### Establishment of the wound model and wound healing analysis

Two full-thickness skin defects at 2 cm in diameter were generated on each side of the dorsum of all 18 rats (30–40 days old) anesthetized by intraperitoneal injection of 3% pentobarbital sodium (30 mg/kg). The wounds were covered with a round homemade hard ring to prevent desiccation. The above wounds were randomly divided into three groups. Empty scaffolds without EpSCs seeded were transplanted to wounds in the empty scaffold group, and EpSC-seeded scaffolds were transplanted to wounds in the EpSC scaffold group. No treatment was applied in the control group. Wounds in the three groups were all covered with transparent dressings (Tegaderm, 3M, Saint Paul, MN, USA).

Wounds were observed and photographed on days 0, 3, 7, 14, and 21. The peripheral rim of the neoskin was excised for further study. Wound areas were measured by ImageJ software v1.53a. The residual wound area rate was calculated as [(day n area)/(day 0 area)] × 100% (n = 0, 3, 7, 14, or 21).

### Statistical analysis

Data are shown as the mean ± standard error of the mean. Comparisons between control and experimental groups at the same time point were conducted by using unpaired two-tailed t test. With more than two groups for multiple comparisons to a control group at different time points, one-way analysis of variance followed by the Bonferroni test was used. All statistical analyses were performed by using SPSS 18.0 software (SPSS, Chicago, IL, USA), and P< 0.05 was considered statistically significant.

## Results

### Characterization of electrospun micro/nano scaffolds and morphological changes of EpSCs on scaffolds

**Fig. 1:**
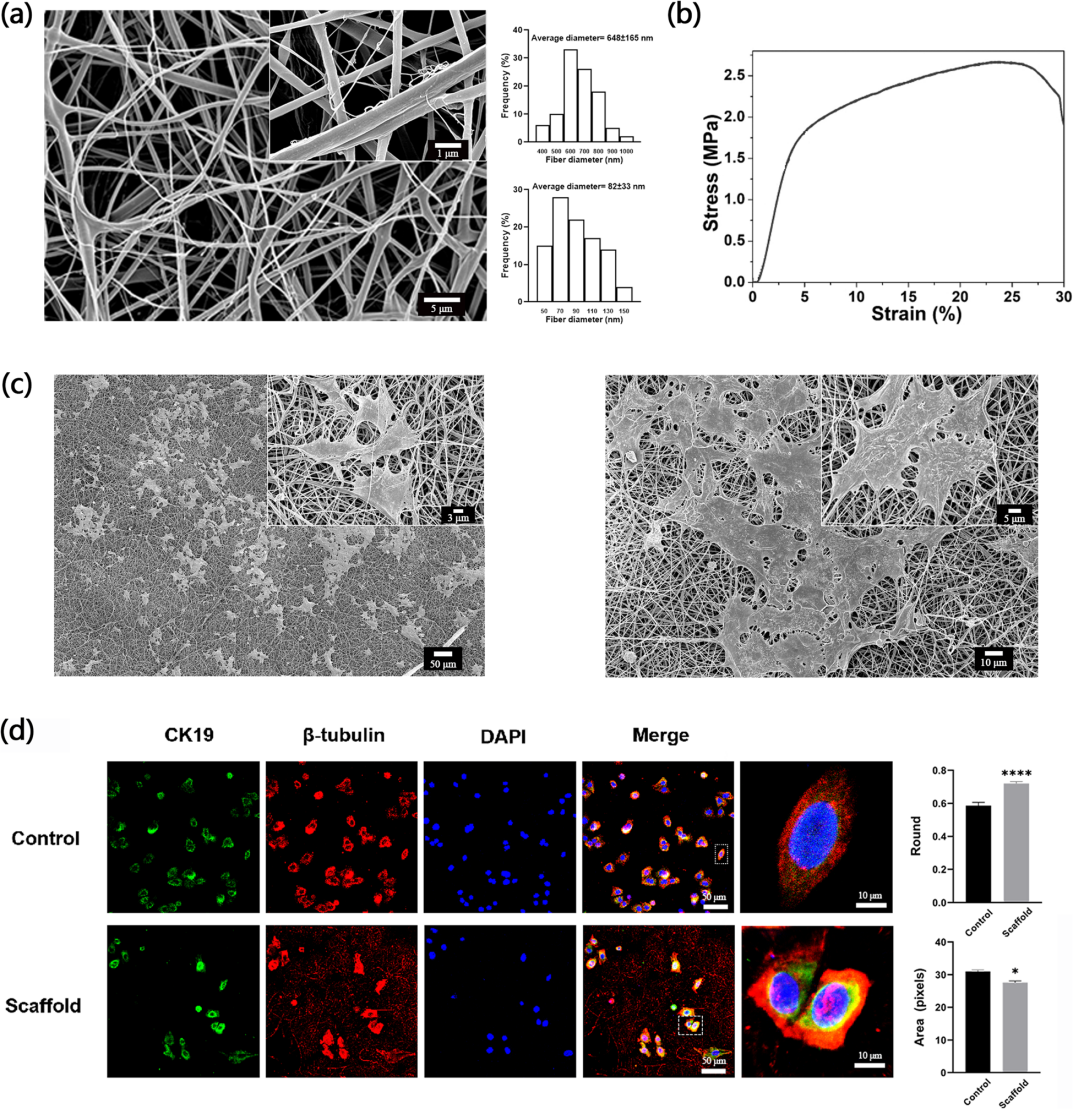
Characterization of electrospun micro/nanoscaffolds and morphological changes in EpSCs on scaffolds. **a** FE-SEM image of the arrangement and diameter distribution of micro/nanofibers in the PCL+CA fiber scaffold. Scale bar = 1 μm, 5 μm. **b** Stress-strain curve of the micro/nanofiber scaffold. **c** FE-SEM images of EpSC-seeded micro/nanofiber scaffolds. Scale bar = 5 μm, 50 μm, 3 μm, 10 μm. **d** Cytokeratin 19 (green) and β-tubulin (red) staining of cells seeded in IV collagen-coated culture dishes and in scaffolds. Scale bar = 50 μm, 10 μm. *P<0.05 and ***P<0.001 vs control group

To characterize the topographic features of the nanofiber scaffold, scanning electron microscopy (SEM) was used to image the 3D structure of the PCL+CA micro/nano scaffold. Apparently, the PCL+CA nanofiber scaffolds have a 3-dimensional porous reticular structure. Of note, the scaffold displayed microfibers (average fiber diameter = 648±165 nm) entangled with thinner nanofibers (average fiber diameter = 82±33 nm) (Fig. 1a). The stress-strain curve was tested and calculated to show the mechanics of the scaffold. As the figure showed, for the scaffold, tensile strength at break 2.65 MPa elongation at break 25.66% (Fig. 1b). To determine whether PCL+CA nanofiber scaffolds are suitable for the adhesion of epidermal stem cells (EpSCs), which is essential for mimicking the in vivo microenvironment, we used SEM to image EpSC-seeded scaffolds. As the images show, EpSCs were tightly attached to the scaffold with full spread, and adjacent cells could connect with each other. Moreover, seeded EpSCs were slightly trapped by micro/nanofiber scaffolds, which partially mimicked the in vivo basal epidermal cell extracellular environment (Fig. 1c). Other characterizations of PCL+CA scaffolds in our laboratory have been documented in previously published articles [7, 13]. To further identify the morphological changes of EpSCs seeded on micro/nanoscaffolds, we used β-tubulin to display the morphological structure of EpSCs and EpSCs marker cytokeratin 19 to mark EpSCs through immunofluorescence staining. EpSCs seeded on IV collagen-coated culture dishes were used as the control group. The results showed that EpSCs seeded on the scaffold were rounder and smaller than those seeded on the control group (Fig. 1d).

### Micro/nanoscaffolds maintain the stemness of EpSCs through inhibition of the Notch signaling pathway

**Fig. 2:**
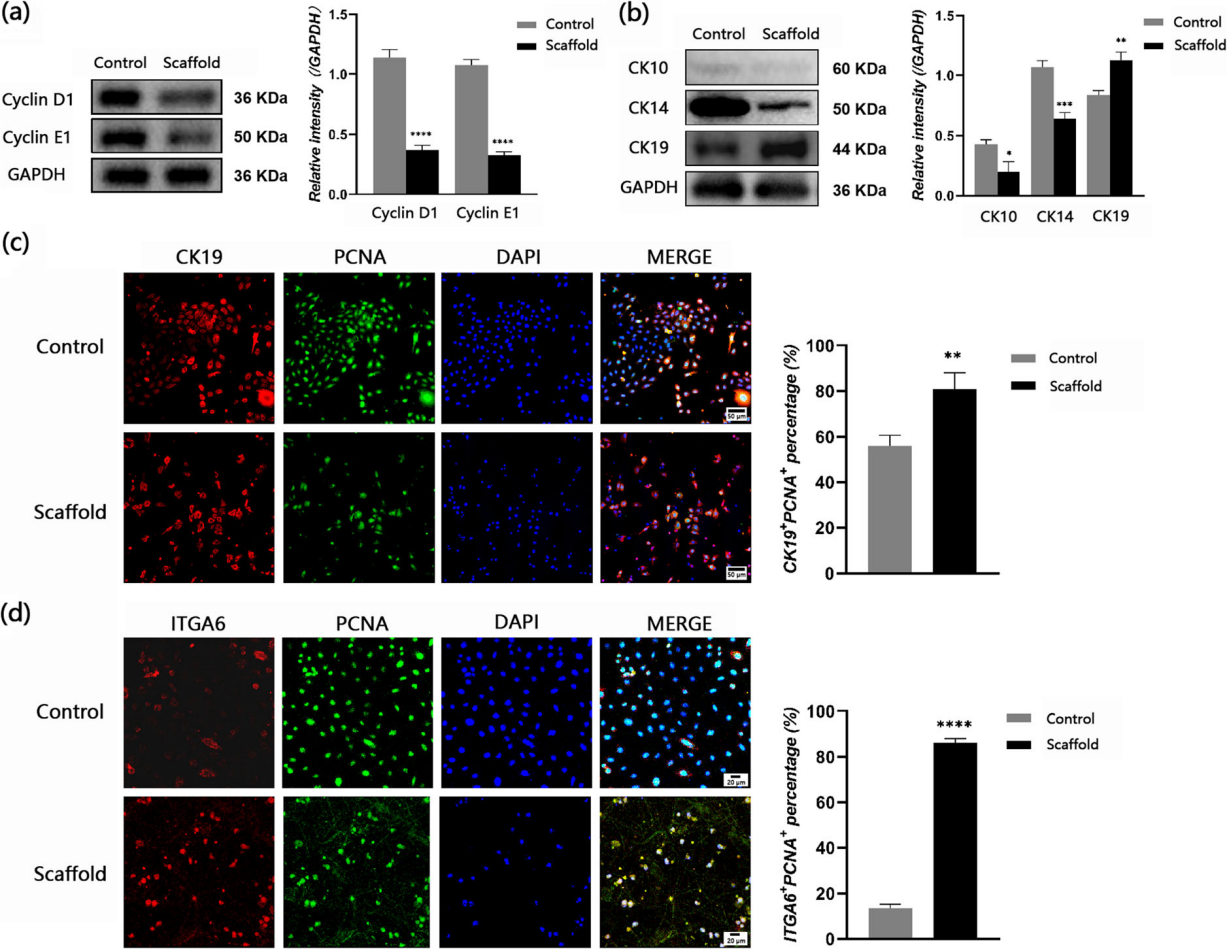
PCL+CA micro/nanofiber scaffold can maintain the stemness of EpSCs through inhibition of the Notch signaling pathway. **a** Representative western blot images and densitometric analysis results showing the relative expression levels of cyclin D1/E1 in EpSCs cultured in 2D dishes and scaffolds. **b** Representative western blot images and results of densitometric analysis showed relative expression levels of ESC differentiation-related proteins cytokeratin 10/14/19 in EpSCs cultured in 2D dishes and scaffolds. **c** Cytokeratin 19 (red) and PCNA (green) staining of cells seeded in IV collagen-coated culture dishes and in scaffolds. Scale bar = 50 μm. **d** Integrin ɑ6 (red) and PCNA (green) staining of cells seeded in IV collagen-coated culture dishes and in scaffolds. Scale bar = 20 μm. *P<0.05, **P<0.01, ***P<0.001, ****P<0.0001 vs the control group

Stemness plays a vital role in the application of EpSCs to repair wounds. To identify the effect of the micro/nanoscaffold on maintaining the stemness of EpSCs, we first detected the expression of the cell cycle-related proteins cyclin D1 and cyclin E1 in EpSCs. EpSCs seeded on IV collagen-coated culture dishes were set as the control group, and those seeded on scaffolds were set as the scaffold group. The results showed decreased expression of cyclin D1 and cyclin E1 in the EpSCs in the scaffold group compared with the control group, which means that fewer EpSCs entered the cell cycle when seeded on the scaffold (Fig. 2a). Then, we detected the expression of differentiation- or stemness-related proteins in EpSCs seeded in different environments. Cytokeratin 10 and 14 are markers of differentiated keratinocytes while cytokeratin 19 is the marker of basal layer EpSCs. The results showed lower expression of cytokeratin 10 and cytokeratin 14 and higher expression of cytokeratin 19 in EpSCs seeded on scaffolds than in EpSCs seeded on IV collagen-coated culture dishes (Fig 2b). To determine whether scaffold microenvironment impairs proliferative potential of EpSCs, we chose PCNA to reflect the proliferation potential of EpSCs. The results of double-immunofluorescence staining of PCNA and cytokeratin 19 showed that the percentage of CK19^+^/PCNA^+^ EpSCs was higher in the scaffold group than in the control group (Fig. 2c). Integrin ɑ6 is a canonical stemness-related marker in EpSCs. The results of double immunofluorescence staining of PNCA and integrin ɑ6 showed that the percentage of ITGA6^+^/PCNA^+^ EpSCs was higher in the scaffold group than in the control group (Fig. 2d).

**Fig. 3:**
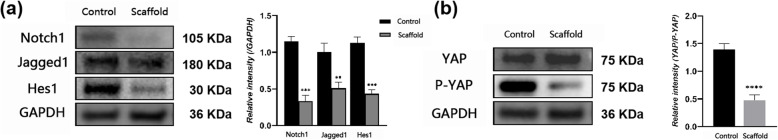
Activation of YAP and inhibition of Notch pathway in EpSCs cultured on scaffold. **a** Representative western blot images and densitometric analysis results showing the relative expression levels of Notch1, Jagged1, and Hes1 in EpSCs cultured in IV collagen-coated culture dishes and scaffolds. **b** Representative western blot images and densitometric analysis results showing the relative expression levels of YAP and P-YAP in EpSCs cultured in IV collagen-coated dishes and scaffolds. **P<0.01, ***P<0.001, ****P<0.0001 vs control group

Notch signaling is involved in modulating the differentiation process of EpSCs in the epidermis, as Notch activation can promote the differentiated state in suprabasal layers, while Notch in basal cells must be somehow inhibited to maintain the stemness of EpSCs. To further explore whether scaffold maintenance of the stemness of EpSCs is related to the inhibition of Notch, we detected the expression of the Notch signaling pathway-related proteins Notch1, Jagged1, and Hes1. The results showed that the expression of these proteins was lower in the scaffold group than in the control group (Fig. 3a). To testify whether activation of YAP was affected which may further cause the suppression of Notch pathway, we detect the activation level of YAP in EpSCs in each group. The results showed that the activation level of YAP (YAP/P-YAP) in scaffold group was remarkably lower compared with in control group (Fig. 3b).

### Characterization of proteomic features of EpSCs cultured on PCL+CA micro/nanofiber scaffolds

**Fig. 4:**
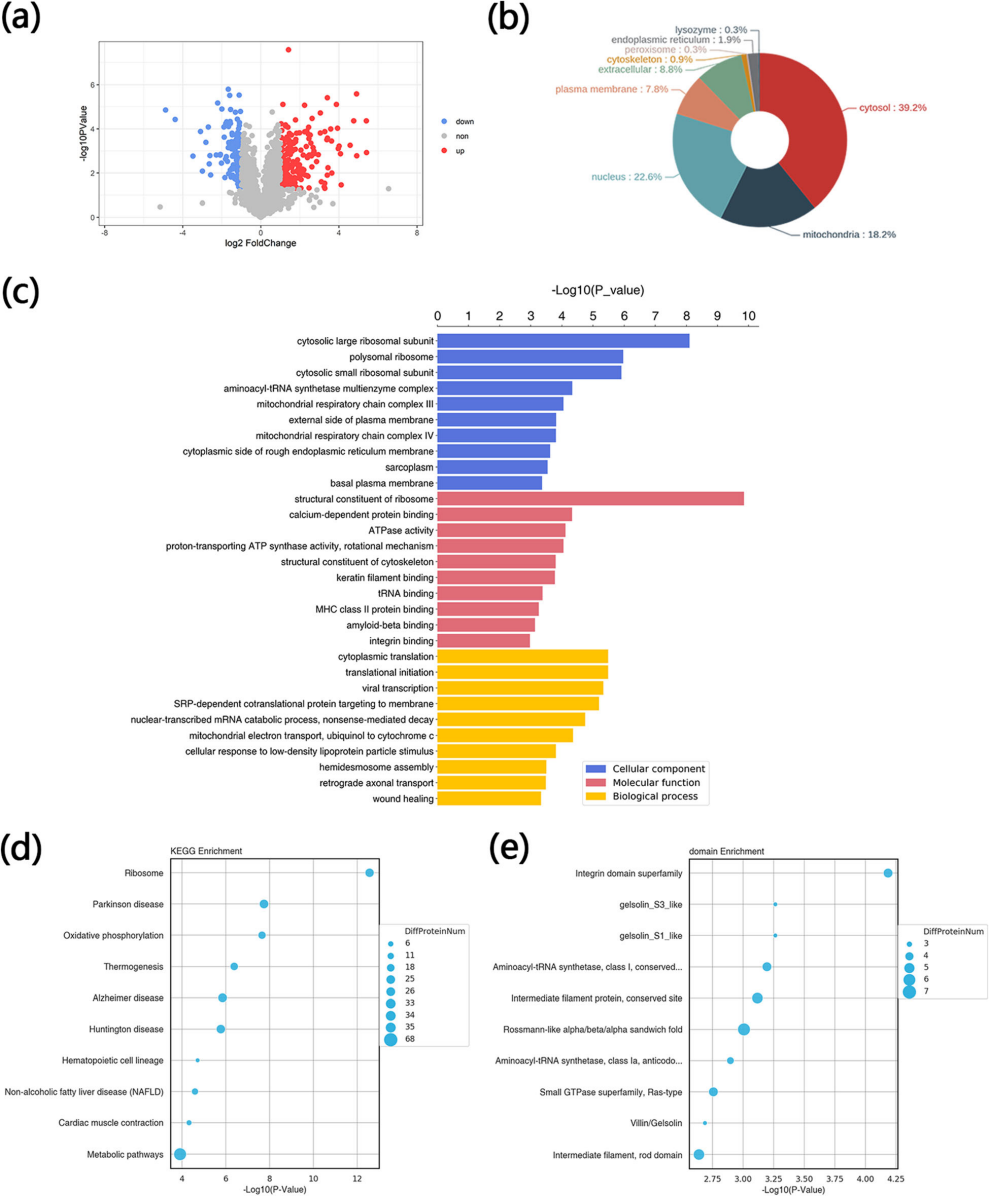
Proteomic analysis of EpSCs cultured in 2D dishes and micro/nanofiber scaffolds. **a** Volcano plot showing proteomic differences in EpSCs cultured in IV collagen-coated culture dishes (control) and micro/nanofiber scaffolds (scaffold group). A p value of <0.05 and a ratio of >2 or <0.5 were considered to indicate a significant difference. **b** Pie plot showing subcellular localization analysis of differentially expressed proteins. **c** GO analysis of differential expression proteins. **d** KEGG analysis of differentially expressed proteins. **e** Enrichment analysis of functional domains of differentially expressed proteins

To further explore proteomic changes in EpSCs after seeding on scaffolds, liquid chromatography-tandem MS (LC-MS/MS) for label-free quantitative proteomics (LFQP) was used to compare the proteomic differences between EpSCs cultured on scaffolds and IV collagen-coated culture dishes (control/scaffold). A total of 3899 proteins were detected by LFQP, and 319 differentially expressed proteins are displayed in a volcano plot (Fig. 4a). A p value of <0.05 and a ratio of >2 or <0.5 were considered to indicate a significant difference. Compared with EpSCs cultured on culture dishes, a total of 124 proteins were upregulated and 195 proteins were downregulated in EpSCs seeded on scaffolds. The subcellular localization of proteins is important information for the study of protein functions. Therefore, WoLF PSORT software was used for the subcellular localization analysis of the differential proteins. The results showed that the cytoplasm, nucleus, and mitochondrion were the top 3 subcellular localizations of differential proteins (Fig. 4b).

To extend the molecular characterization of differentially expressed proteins, UniProt and GO databases were used to characterize information about the terms biological process (BP), molecular function (MF), and cellular component (CC). The detailed GO annotation distribution of differential proteins is displayed in a histogram divided into three groups (BP, MF, and CC). Fisher’s exact test was used to evaluate the significance level of single GO term protein enrichment (Fig. 4c). The top 10 most significantly enriched GO term classifications are listed.

With the KEGG pathway as the unit and all qualitative proteins as the background, Fisher’s exact test was used to analyze and calculate the significance level of protein enrichment of each pathway to determine the metabolism and signal transduction pathways that were significantly affected between the two groups. The top 10 KEGG enrichment pathways are displayed in the bubble diagram (Fig. 4d).

Proteins are composed of structural domains, which are units of protein structure, function, and evolution. The study of protein domains is of great significance for understanding the biological functions and evolution of proteins. InterPro is a resource database that combines protein families, domains, and functional sites. We used this database to analyze the enrichment of functional domains of differential proteins. The top 10 classification results with the most significant enrichment are shown in the bubble diagram (Fig. 4e).

### EpSC-seeded micro/nanoscaffolds can promote wound healing through activation of the Notch signaling pathway

**Fig. 5:**
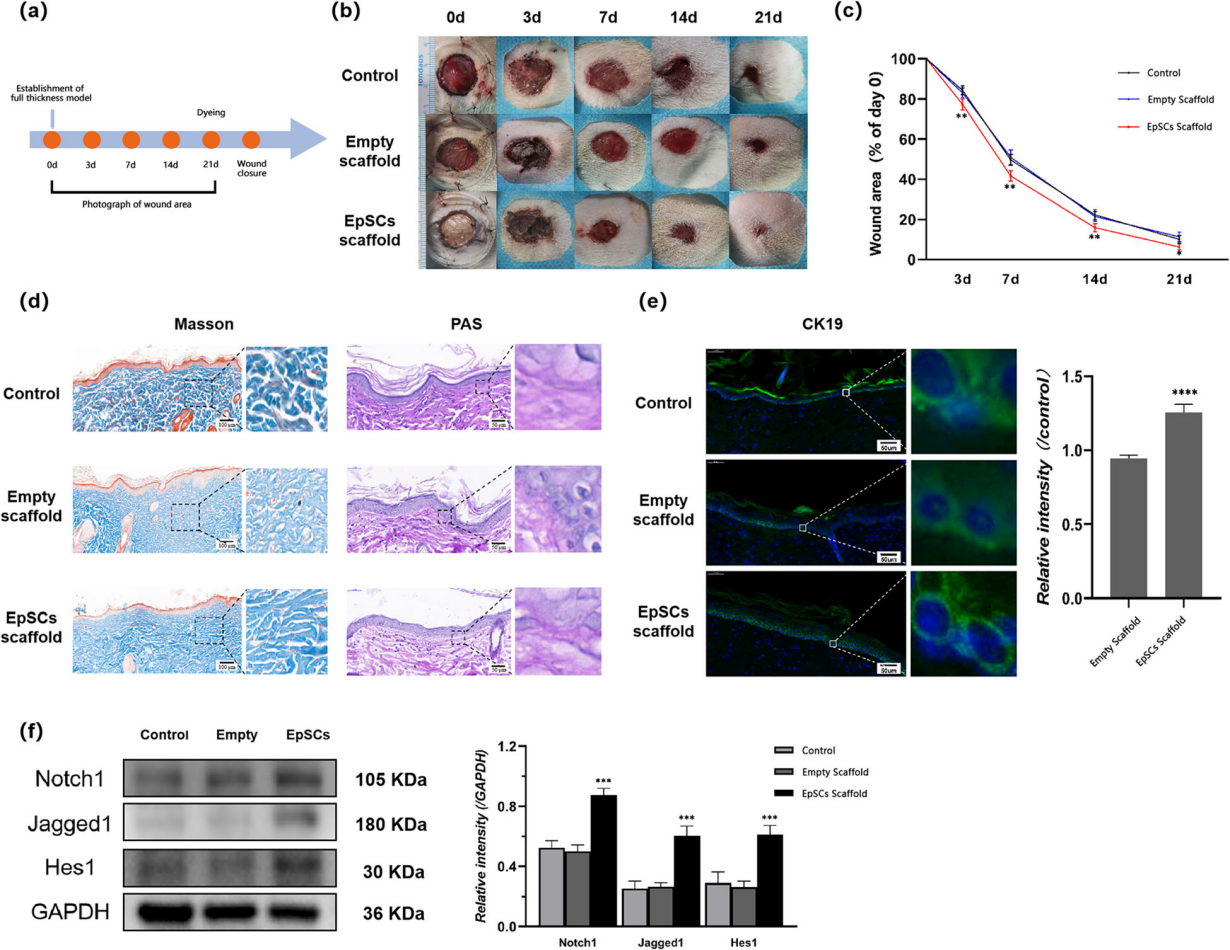
EpSC-seeded PCL+CA micro/nanofiber scaffolds can promote wound healing through activation of the Notch pathway. **a** Process map of whole animal experiment. **b** Representative images of wounds from each group taken at 0 day, 3 days, 7 days, 14 days, and 21 days post-injury. **c** Wound areas are shown for the control group, empty scaffold group, and EpSC scaffold group wounds. The wound healing rate was calculated by dividing the current area by the initial area. **d** Masson staining and PAS staining of neoskin taken 21 days post-injury. Scale bar = 100 μm (Masson staining), 50 μm (PAS staining). **e** Immunofluorescence staining of cytokeratin 19 (green) in neoskin taken 21 days post-injury. Scale bar = 50 μm. **f** Representative western blot images and densitometric analysis results showing the expression levels of the Notch pathway-related proteins Notch1, Jagged1, and Hes1 in neoskin sampled 21 days postinjury from each group. *P<0.05, **P<0.01, ***P<0.001 vs control group

During wound repair, EpSCs play a vital role by providing a source for replenishing lost cells [17, 18]. Moreover, they can provide growth factors to promote angiogenesis during wound healing [19]. To explore whether stemness-maintained EpSCs can promote wound healing, we used EpSC-seeded scaffolds for transplantation in a SD rat full-thickness skin defect model to observe their roles in wound healing (Fig. 5a).

Compared with the control group, the empty scaffold-transplanted group did not impede wound healing, and the scaffold was degraded within 7 days. In addition, wound healing in the EpSCs scaffold-transplanted group was significantly accelerated compared with that in the control group and was nearly completely healed in 21 days (Fig. 5b,c). To further evaluate the quality of wound healing, we observed the generation and deposition of collagen and the formation and integrity of the basement membrane in neoskin around wound areas by Masson staining and PAS staining. The staining results showed that the arrangement of collagen was more regular and ordered in the EpSCs scaffold-transplanted group than in the control group and empty scaffold-transplanted group. In addition, the results of PAS staining showed a more intact basement membrane in the EpSCs scaffold-transplanted group than in the other groups (Fig. 5d). The viability of EpSCs in neoskin around wound areas was also detected by immunofluorescence staining of cytokeratin 19. The results showed that keratinocytes in the basal layer of neoskin exhibited a higher expression of cytokeratin 19 in EpSCs scaffold transplantation than in the control group (Fig. 5e).

The Notch signaling pathway is involved in wound healing, and activation of the Notch signaling pathway can promote wound healing. To explore the mechanism underlying the promotion of wound healing in the EpSCs scaffold-transplanted group, we detected the expression of the Notch signaling pathway-related proteins Notch1, Jagged1, and Hes1 in skin tissue by western blot. The results showed that the expression of Notch1, Jagged1, and Hes1 in the skin tissue in the ESC scaffold-transplanted group was higher than that in the other groups, which indicated that the notch signaling pathway in the ESC scaffold-transplanted group was activated (Fig. 5f).

## Discussion

The topographic features of electrospun scaffolds play a vital role in modulating the biological functions of cells seeded on the scaffolds [20–22]. The impact of different topographic features of the extracellular environment on the morphology of cells is related to cell mechanics, which further modulate cell morphology. In our former study, we demonstrated that micro/nanofiber scaffolds could partially simulate the in vivo function of the basement membrane [10]. In vivo, the basement membrane can modulate the biological behavior of EpSCs, as EpSCs sense the extracellular microenvironment to change their patterning [23, 24]. Inhibition of the differentiation of EpSCs needs to restore extracellular cues provided by the stem cell niche [25, 26]. Thus, we used a PCL+CA micro/nanofiber scaffold mimicking the basement membrane to culture EpSCs to observe the modulation effect of the scaffold on EpSCs. In this study, we found that the morphology of EpSCs was smaller and rounder in scaffolds than in culture dishes. The morphology of EpSCs is implicated in differentiation because EpSCs exhibit different differentiation statuses in substrates with different topographies [27, 28]. Herein, we speculated that the unique topographic features of the PCL+CA micro/nanofiber scaffold mimicking the extracellular basement membrane structure contribute to the specific cell morphological changes in EpSCs, which may further affect the stemness of EpSCs. Further study of specific topographic features that directly modulate the differentiation of EpSCs in PCL+CA micro/nanofiber scaffolds needs to be performed in our following work.

The maintenance of the stemness of EpSCs restricts their application, as the enrichment level of EpSCs in cultured epidermal cell sheets influences the outcome of wound repair [29]. In our study, we found that the stemness of EpSCs could be maintained when cultured on a PCL+CA electrospinning micro/nanofiber scaffold. The reduced expression of cyclin D1/E1 indicated that EpSCs in the scaffold undergo a slower cell cycle than those cultured on dishes, which may contribute to the stemness maintenance of EpSCs. Although the differentiation of EpSCs was inhibited on the scaffold, the proliferation ability of EpSCs was not impaired because the expression level of PCNA in EpSCs cultured on the scaffold was similar to that in EpSCs cultured on dishes. In addition, we found that YAP is activated in EpSCs cultured on scaffolds, while the Notch signaling pathway involved in the regulation of differentiation in EpSCs is inhibited. YAP/TAZ is linked to cell mechanics changes to modulate the Notch pathway for the control of epidermal stem cell fate, as the activation of YAP/TAZ can inhibit the Notch pathway [12]. Thus, we considered that the scaffold maintains the stemness of EpSCs through activation of YAP/TAZ, which in turn inhibits the Notch signaling pathway to suppress the differentiation of EpSCs.

The application of biomaterial scaffolds for culturing EpSCs is universal in tissue engineering [30, 31]. However, few studies have used mass spectrometry to systematically analyze the proteomic features of EpSCs to further uncover overall biological changes in EpSCs cultured on scaffolds. In this study, we used MS to analyze proteomics in EpSCs cultured on micro/nanofiber scaffolds and culture dishes. Significant proteomics changes were observed in EpSCs cultured on scaffolds compared with EpSCs cultured on dishes. Among the domain structures of the differentially expressed proteins, the integrin domain superfamily differs most significantly. Integrin-mediated adhesions have long been acknowledged to provide the pivotal molecular link attaching cells to the extracellular matrix and serve as a bridge to transmit signals between cells and the extracellular microenvironment [32]. Our results indicated that the modulation of EpSCs by scaffolds depends on integrin-mediated signal transduction. Notably, in the GO analysis and KEGG analysis, the expression of ribosome-related proteins was differential in EpSCs between the scaffold and control groups. However, whether they participate in modulating the differentiation of EpSCs was not elucidated in this study. Interestingly, we also found that the expression of metabolic process-related proteins differed in EpSCs cultured on different substrates. Glycolysis can respond to architectural features of the actomyosin cytoskeleton, which is remodeled after sensing changes in the extracellular environment [33]. Our findings also correspond to the linking between cell metabolism and the mechanical properties of the extracellular microenvironment. Thus, further exploration of the mechanism by which cell metabolism changes modulate the stemness changes of EpSCs and the relations between ribosome-related proteins and differentiation modulation needs to be carried out in our next plan.

The mechanism underlying the healing promotion of transplanted allogeneic EpSCs during wound healing involves many signaling pathways [34]. In this study, we found that ESC-transplanted wounds exhibited an accelerated speed of healing and activation of the Notch signaling pathway. Under certain conditions, autologous transplanted EpSCs can differentiate into vascular endothelial cells to promote angiogenesis and wound healing [19]. Our findings indicated that allogeneic EpSCs may promote wound healing by indirectly secreting cytokines that could activate the Notch signaling pathway. The Notch and Wnt signaling pathways are involved in the wound healing process [35]. However, in this study, we did not elucidate whether the Wnt/β-catenin signaling pathway is coactivated to promote wound healing, which needs further experimental testing.

In summary, in our study, we used an electrospinning technique to build a PCL+CA micro/nanofiber scaffold that is suitable for the culture of EpSCs. Exploration of the modulation effect of the prepared scaffold on EpSCs showed that the PCL+CA micro/nanofiber scaffold can maintain the stemness of EpSCs in vitro through the inhibition of the Notch signaling pathway by activating YAP. Further proteomics analysis of scaffold-cultured or IV collagen-coated culture dish-cultured EpSCs showed distinct changes in proteomics between EpSCs cultured on two different substrates, and changes in metabolic pathway-related proteins were particularly observed. Moreover, the transplantation of stemness-maintained EpSCs by the PCL+CA micro/nanoscaffold in rat full-thickness defect models accelerated wound healing through activation of the Notch pathway.

## Conclusions

In our study, we demonstrated that the PCL+CA electrospun micro/nanofiber scaffold can maintain the stemness of EpSCs in vitro through inhibition of the Notch signaling pathway. Using mass spectrometry technology, we systematically analyzed the proteomic changes in EpSCs cultured on scaffolds compared with EpSCs with lost in vitro stemness and found that the expression levels of ribosome-related proteins and metabolic pathway-related proteins were significantly changed. Moreover, we demonstrated that stemness-maintained EpSCs transplanted with the PCL+CA scaffold could promote wound healing through activation of the Notch signaling pathway. Overall, our study indicated that the PCL+CA electrospun micro/nanofiber scaffold is capable of establishing an in vitro stem cell niche microenvironment, which is helpful for further exploration of the mechanism underlying stemness modulation ex vivo. In addition, the PCL+CA scaffold could be a new option for the transplantation of undifferentiated EpSCs to promote wound healing.

## Data Availability

The datasets analyzed during the current study are not publicly available due to that some experiments are still in progress but are available from the corresponding author on reasonable request.

## Abbreviations

EpSC: Epidermal stem cell
PCL: Polycaprolactone
CA: Cellulose acetate
LC-MS/MS: Liquid chromatography-tandem mass spectrometry
LFQP: Label-free quantitative proteomics
FE-SEM: Field emission scanning electron microscopy
SD: Sprague–Dawley
BP: Biological process
MF: Molecular function
CC: Cellular component
